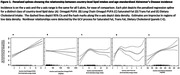# Alzheimer disease incidence increases with country‐level intake of Omega‐6 Polyunsaturated Fatty Acids

**DOI:** 10.1002/alz.093189

**Published:** 2025-01-09

**Authors:** Timothy H Ciesielski, Razaq O Durodoye, Giuseppe Tosto, Margaret A Pericak‐Vance, Jonathan L. Haines, Alzheimer’s Disease Genetics Consortium (ADGC), Scott M Williams

**Affiliations:** ^1^ Case Western Reserve University, Cleveland, OH USA; ^2^ Taub Institute for Research on Alzheimer’s Disease and the Aging Brain, Vagelos College of Physicians and Surgeons, Columbia University, New York, NY USA; ^3^ 1501 NW 10th Avenue, Miami, FL USA; ^4^ Department of Population and Quantitative Health Sciences, Institute for Computational Biology, Case Western Reserve University, Cleveland, OH USA; ^5^ Department of Population and Quantitative Health Sciences, Institute for Computational Biology, Case Western Reserve University, Cleveland, PA USA

## Abstract

**Background:**

Evidence from diverse fields implicates lipid dysfunction in Alzheimer Disease (AD) pathogenesis. However, lipid consumption at the individual level does not vary greatly within most study cohorts, and multiple lipids are rarely well‐measured concurrently. In contrast, mean lipid intakes at the country‐level can be precisely estimated and can vary substantially across countries. These can be compared to annual age‐standardized Alzheimer Disease incidence (ASAIR) to assess the role of lipid consumption in population risk.

**Methods:**

Mean country‐level lipid intake estimates were compared to ASAIR in 183 countries across all 6 inhabited continents. Penalized spline regression analysis including a lag between intake and incidence was used to assess nonlinearities in the relationships between 5 lipids and ASAIR. We then used multivariable‐adjusted linear regression models to quantify the linear portions of these relationships. Validation was conducted using longitudinal within‐country changes between 1990 and 2019.

**Results:**

Bivariate penalized spline regression analyses revealed non‐linearity in the relationships between saturated‐fat, trans‐fat, dietary cholesterol and ASAIR. For each lipid, ASAIR increased with increasing intake up to a threshold, however in the lower‐powered longitudinal validation attempts these associations in the low‐intake range were not significant. Omega3 and Omega6 Polyunsaturated‐Fatty‐Acids (PUFA) exhibited linear relationships between intake and ASAIR in the spline analyses. The multivariable‐adjusted linear regression model revealed a positive association between Omega6‐PUFA (as a % of total energy intake) and ASAIR (β = 2.44; 95%CI: 1.70, 3.19; p = 1.38×10^−9^). Longitudinal analyses comparing the percent change in the country‐level intake from 1990 to 2010 to the percent change in ASAIR from 1990 to 2019 further supported the direction and significance of this relationship (β = 0.036; 95%CI: 0.015, 0.057; p = 8.21×10^−4^).

**Conclusions:**

Our results indicate that higher levels of Omega6 PUFA consumption associate with increased incidence of AD globally. From a public health point of view, national recommendations or policy to decrease Omega6 PUFA consumption could have substantial benefits in reducing the burden of AD. The scale of benefits will vary by country, but in the US alone our results predict that a 2 SD decrease in mean Omega‐6 PUFA intake would reduce ASAIR by 8% (∼28,000 incident cases).